# Alkane Biosynthesis Genes in Cyanobacteria and Their Transcriptional Organization

**DOI:** 10.3389/fbioe.2014.00024

**Published:** 2014-07-14

**Authors:** Stephan Klähn, Desirée Baumgartner, Ulrike Pfreundt, Karsten Voigt, Verena Schön, Claudia Steglich, Wolfgang R. Hess

**Affiliations:** ^1^Genetics and Experimental Bioinformatics, Institute of Biology 3, Faculty of Biology, University of Freiburg, Freiburg, Germany

**Keywords:** alkane biosynthesis, start sites of transcription, cyanobacteria, operon, promoter, sRNA

## Abstract

In cyanobacteria, alkanes are synthesized from a fatty acyl-ACP by two enzymes, acyl–acyl carrier protein reductase and aldehyde deformylating oxygenase. Despite the great interest in the exploitation for biofuel production, nothing is known about the transcriptional organization of their genes or the physiological function of alkane synthesis. The comparison of 115 microarray datasets indicates the relatively constitutive expression of *aar* and *ado* genes. The analysis of 181 available genomes showed that in 90% of the genomes both genes are present, likely indicating their physiological relevance. In 61% of them they cluster together with genes encoding acetyl-CoA carboxyl transferase and a short-chain dehydrogenase, strengthening the link to fatty acid metabolism and in 76% of the genomes they are located in tandem, suggesting constraints on the gene arrangement. However, contrary to the expectations for an operon, we found in *Synechocystis* sp. PCC 6803 specific promoters for the two genes, *sll0208* (*ado*) and *sll0209* (*aar*), which give rise to monocistronic transcripts. Moreover, the upstream located *ado* gene is driven by a proximal as well as a second, distal, promoter, from which a third transcript, the ~160 nt sRNA SyR9 is transcribed. Thus, the transcriptional organization of the alkane biosynthesis genes in *Synechocystis* sp. PCC 6803 is of substantial complexity. We verified all three promoters to function independently from each other and show a similar promoter arrangement also in the more distant *Nodularia spumigena*, *Trichodesmium erythraeum*, *Anabaena* sp. PCC 7120, *Prochlorococcus* MIT9313, and MED4. The presence of separate regulatory elements and the dominance of monocistronic mRNAs suggest the possible autonomous regulation of *ado* and *aar*. The complex transcriptional organization of the alkane synthesis gene cluster has possible metabolic implications and should be considered when manipulating the expression of these genes in cyanobacteria.

## Introduction

The production of liquid fuels and a variety of chemicals indispensable for daily life depends on fossil resources. Due to their limited availability and with respect to environmental concerns the exploitation of alternative, renewable, and sustainable energy sources is gaining in importance. Thus, the conversion of solar energy and carbon dioxide into biofuels and suitable chemicals, e.g., hydrogen, ethanol, ethylene, isobutyraldehyde, or isoprene by genetically modified and improved phototrophic microorganisms such as cyanobacteria is of high interest (Deng and Coleman, 1999; Takahama et al., 2003; Atsumi et al., 2009; Lindberg et al., 2010; McKinlay and Harwood, 2010; Georgianna and Mayfield, 2012; Peralta-Yahya et al., 2012).

Interestingly, many cyanobacteria are naturally able to produce alkanes (Winters et al., 1969), which are the major constituents of gasoline, diesel, and jet fuels. However, there are also strains such as *Synechococcus* sp. PCC 7002 in which no alkanes were detectable, indicating that the responsible synthesis pathway is not present in these strains. Considering this information and using a comparative genomics approach, the according genes were identified (Schirmer et al., 2010). In cyanobacteria, alkanes are synthesized from intermediates of the fatty acid metabolism (Figure S1 in Supplementary Material) by two enzymes: acyl–acyl carrier protein reductase (AAR) and aldehyde deformylating oxygenase (ADO) (Schirmer et al., 2010; Li et al., 2011, 2012; Zhang et al., 2013). These enzymes are encoded by the two adjacent genes *sll0208* (*ado*) and *sll0209* (*aar*) in the model strain *Synechocystis* sp. PCC 6803 (from here: *Synechocystis* 6803). Interestingly, orthologs of these genes have been found so far only in cyanobacteria, suggesting the possible existence of a link to photoautotrophic life style i.e., oxygenic photosynthesis, but the functional relevance of cyanobacterial alkane biosynthesis *in vivo* has remained enigmatic thus far.

Cyanobacteria are morphologically very complex and are represented by unicellular (e.g., *Synechocystis* 6803, *Prochlorococcus* sp. MIT9313) as well as multicellular strains with differentiated cells (e.g., *Anabaena* sp. PCC 7120, *Nodularia spumigena* sp. CCY9414). Moreover, cyanobacteria are found in nearly all light-exposed habitats on earth including extreme environments such as deserts (Cameron, 1962; Garcia-Pichel et al., 2001), hot springs (Miller and Castenholz, 2000), hypersaline water (Reed et al., 1984) as well as Antarctic meltwater ponds (Nadeau and Castenholz, 2000), which underlines also their physiological diversity. Based on morphological properties five subsections were defined. However, due to the insufficient coverage of the cyanobacterial phylum by full genome sequences, truly comprehensive genome analyses have remained impossible for a long time. Recently, 54 additional genomes have been published (Shih et al., 2013), overcoming the lack of genomic data and enabling a comprehensive view on the occurrence and organization of genes important for a particular environmental situation.

For many cyanobacterial strains the expression of *ado* and *aar* orthologs is evident since long-to-medium-chain alkanes were detected (Winters et al., 1969; Schirmer et al., 2010). Though, their physiological function as well as the regulation of expression remains elusive. In *Synechocystis* 6803, alkane synthesis could be abolished by deletion of *ado*/*aar* and thus appears not essential, at least under standard growth conditions (Schirmer et al., 2010). To make an impact on cell physiology, genes need to be expressed. Furthermore, if their function is only needed under certain conditions, their expression should be regulated. The presence of upstream genetic regulatory elements can to a great extent serve as evidence for the functional significance of a gene. Thus, a detailed expression analysis might point to certain environmental conditions under which alkane synthesis is physiologically more relevant or even essential. Global transcriptomic analyses using microarrays are powerful approaches to investigate gene regulation and comparative data for manifold environmental conditions are available also for cyanobacteria (Hernandez-Prieto and Futschik, 2012). Moreover, RNA-seq, especially the differential RNA sequencing approach [dRNAseq, (Sharma et al., 2010)] is often used for the analysis of the primary transcriptome, which provides insight into gene expression changes together with detailed information about transcriptional start sites (TSS) and all promoters active at a given moment. By now, the primary transcriptomes of several cyanobacteria are available, supporting the analysis of the transcriptional organization of alkane synthesis (Mitschke et al., 2011a,b; Voß et al., 2013; Voigt et al., 2014; Pfreundt et al., submitted).

In this work, we present a comprehensive analysis of the genomic arrangement of genes encoding ADO and AAR throughout the cyanobacterial phylum. Moreover, we investigated the transcriptional organization of these genes for *Synechocystis* 6803 but also for *Anabaena* PCC 7120, *N. spumigena* CCY9414, *Prochlorococcus* MIT9313 & MED4, and *Trichodesmium erythraeum* IMS101. Although a dicistronic or polycistronic arrangement appears conserved at the genomic level among almost all cyanobacteria, we found solid evidence for the independent transcription of *ado* and *aar* in all tested strains. Since these strains are rather distantly related, the data indicate that an independent transcription of both genes might be common also for most other cyanobacteria. Additionally, for *Synechocystis* 6803 we compiled available expression data extracted from 115 microarray datasets, which comprise more than 25 environmental stimuli but reveal only modest conditional changes in gene expression.

## Materials and Methods

### Cluster analysis

By using the JGI database and blastP algorithm (threshold *E*-value = 1e^−5^), 181 cyanobacterial genomes were screened for orthologs of ADO and acyl-ACP reductase genes. The full list of all genomes included in the study is shown in Table S1 in Supplementary Material. The corresponding protein sequences from *Synechocystis* 6803 were used as reference. For acetyl-CoA carboxylase, short-chain dehydrogenase, and GTP cyclohydrolase I, the respective sequences from *Anabaena* sp. PCC 7120 were used. The phylogenetic tree was generated with MEGA V6.0 (Tamura et al., 2013) by using the neighbor joining algorithm based on 16S rRNA sequences that were extracted from the SILVA database (Quast et al., 2013).

### Strains and growth conditions

The following strains were used: *Synechocystis* 6803, substrain “PCC-M” (Trautmann et al., 2012), *T. erythraeum* IMS101 (obtained from Ilana Berman-Frank, Bar-Ilan University, Tel-Aviv, Israel; originally isolated by Prufert-Bebout et al., 1993) and *Prochlorococcus* MIT9313 & MED4 (courtesy of Sallie W. Chisholm, Massachusetts Institute of Technology, Cambridge, USA). *Synechocystis* was grown in TES-buffered (20 mM, pH 8.0) BG11 medium (Rippka et al., 1979) at 30°C under continuous white light illumination of 50–80 μmol quanta m^−2^ s^−1^ and gentle agitation. To determine the stability of transcripts, cultures were additionally aerated with ambient air through a glass tube and a sterile filter for constant and fast growth. Rifampicin was added as an inhibitor of transcription at a final concentration of 300 μg/ml. Samples were taken before and 3, 5, 10, 15 min after the treatment. *T. erythraeum* cultures were grown in YBCII medium (Chen et al., 1996) at 25°C and 12/12 h light/dark cycles at ~80 μmol photons m^−2^ s^−1^ white light. *Prochlorococcus* cells were grown at 22°C in AMP1 medium (Moore et al., 2007) under 10–30 μmol quanta m^−2^ s^−1^ continuous white cool light. For DNA cloning the *E. coli* strains Top10F′ and DH5α were used and cultivated in LB medium at 37°C.

### RNA extraction, northern blots, and mapping of RNA 5′ and 3′ ends

Cyanobacterial cells were harvested in exponential growth phase by vacuum filtration on hydrophilic polyethersulfone filters (Pall Supor-800, 0.8 μm or Supor-450, 0.45 μm), immediately immersed in 1 ml of PGTX solution (Pinto et al., 2009) and frozen in liquid nitrogen. Total RNA was extracted as described (Hein et al., 2013); *T. erythraeum* and *Prochlorococcus* cells were subjected to bead beating (bead diameter 0.1–0.5 mm) for 3 × 20 s at 6500 rpm (Precellys, Peqlab, Germany) immediately prior to extraction. For northern blot analysis, 3 (*Synechocystis*) to 5 μg (*T. erythraeum* and *Prochlorococcus*) of total RNA were separated on 1.5% agarose gels, transferred to Hybond-N^+^ nylon membranes (GE Healthcare) by capillary blotting and cross-linked by UV-illumination (125 mJ). Generation of single-stranded radioactively labeled RNA probes and hybridization with the blotted RNA were performed as described before (Steglich et al., 2008). The sequences of oligonucleotides used to amplify the respective probe templates are shown in Table S6 in Supplementary Material. Signals were visualized by using a Personal Molecular Imager FX system and Quantity One software (Bio-Rad). The half-life of transcripts was calculated after densitometric quantification of the corresponding signals. Mapping of RNA 5′ and 3′ ends was performed by rapid amplification of cDNA ends as described (Argaman et al., 2001). To define precise lengths, RNA molecules were self-ligated, reverse transcribed using gene-specific primers, and amplified by circular PCR as described (Vogel and Hess, 2001). All used RNA and DNA oligonucleotides are listed in Table S6 in Supplementary Material. Reverse transcription was performed at 42°C for 2 h using the Omniscript™ RT system (Qiagen). Prior to sequencing the PCR products were cloned into the pGEM^®^-T vector (Promega).

### Generation of reporter strains

The putative promoter elements were fused to a reporter gene by PCR amplification (for oligonucleotides see Table S6 in Supplementary Material), followed by restriction digest with *Fse*I/*Age*I and cloning into the promoter probe vector pILA (Kunert et al., 2000). The pILA plasmid contains a promoterless *luxAB* operon encoding subunits of the luciferase enzyme and sequences for the homologous recombination of the entire promoter probe cassette into the *Synechocystis* 6803 gene *slr0168* on the chromosome. The vector used in this work was modified prior to promoter insertion by introducing the restriction recognition sites *Fse*I and *Age*I upstream of the *luxAB* genes. The sites were introduced by PCR amplification of the original pILA plasmid with the primers “Plux *Age*I fw”/“Plux-bla *Age*I rev” and with the primers “Plux-bla *Fse*I fw”/“Plux *Fse*I rev”, resulting in two blunt ended products, which were subsequently ligated. The plasmid derivatives were used for transformation of a *Synechocystis* 6803 strain carrying the *luxCDE* operon in turn encoding enzymes for the synthesis of decanal, the substrate for the luciferase reaction. The expression cassette harboring the genes *luxCDE* under control of the strong promoter of the ncRNA Yfr2a (Voß et al., 2009) and a *cat* gene mediating resistance to chloramphenicol, was integrated into the intergenic region of *sll1691* and *slr1819* (both hypothetical proteins on the chromosome), which is regarded as a neutral site. Transformation was performed as described (Kunert et al., 2000). Genetically modified cells were initially selected on agar-solidified BG11 medium (0.9% KobeI agar, Roth, Germany) containing 10 μg ml^−1^ kanamycin (Km, selection of *luxAB* constructs) and 2 μg ml^−1^ chloramphenicol (Cm, marker for *luxCDE* cassette), but the segregation of clones and subsequent cultivation of mutants was performed in presence of 50 μg ml^−1^ Km and 10 μg ml^−1^ Cm.

### Luciferase assays

Bioluminescence was measured *in vivo* as total light counts per second by using a VICTOR^3^ multiplate reader (PerkinElmer). The cells were grown in the presence of 10 mM glucose. Prior to the measurement, cells were diluted to an OD_750_ = 0.4 and 200 μl of the suspension were filled into a white 96-well plate (CulturePlate™-96, PerkinElmer). In the multiplate reader the cell suspensions were shaken for 10 s and subsequently total light emission was measured for 1 s. A strain carrying the promotorless *luxAB* genes served as a negative control.

## Results

### Occurrence and genomic organization of alkane synthesis genes in cyanobacteria

**Figure 1:**
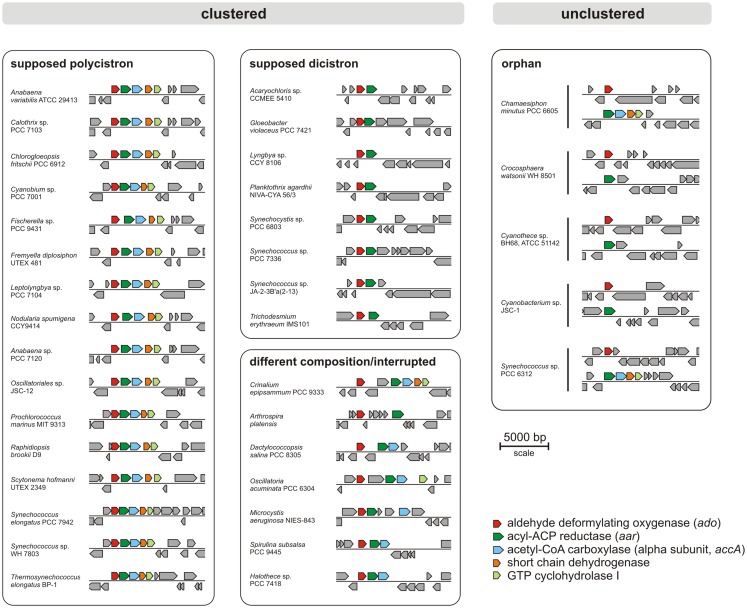
**Examples for genomic arrangements of aldehyde deformylating oxygenase orthologs in cyanobacteria**. By using JGI database and blastP algorithm (threshold *E*-value = 1e^−5^) 181 cyanobacterial genomes were screened for the presence of alkane biosynthesis genes. The corresponding protein sequences from *Synechocystis* sp. PCC 6803 were used as reference.

Today, many cyanobacterial genomes are available in the databases enabling the possibility of comparing genomes with regard to physiological properties such as the production of alkanes. In this study, 181 genomes (Table S1 in Supplementary Material) were screened for orthologs of AAR and ADO (Schirmer et al., 2010; Li et al., 2011, 2012; Zhang et al., 2013). By using the blastP algorithm at an *E*-value cut off 1e^−5^, orthologs of both enzymes could be identified in 90% (163/181) of the genomes (Tables S2 and S3 in Supplementary Material), which underlines their importance. Notably, their functional connection is highlighted by the fact that we found not a single genome in which one of the two genes, *aar* and *ado*, would have been retained in the absence of the other. Moreover, the genomes were also analyzed for possible synteny at the locus encoding ADO. The different types of arrangements are shown in Figure 1. Interestingly, in 76% (138/181) of the genomes tested, the genes encoding AAR and ADO were found adjacent to each other, indicating a possible operon-like organization. Moreover, in 61% (111/181) of the strains both genes clustered together with additional genes encoding the alpha subunit of acetyl-CoA carboxyl transferase (EC 6.4.1.2) and a short-chain dehydrogenase, strengthening the functional connection to fatty acid metabolism. In most genomes, additionally a gene encoding a GTP cyclohydrolase I (EC 3.5.4.16), which is involved in folate biosynthesis, was found downstream of the other four. However, in a few strains *aar* and *ado* are located at different loci as single genes (Figure 1). Additionally, in 18 of the tested genomes AAR and ADO genes were not found (for the full list see Table S4 in Supplementary Material), including the previously studied *Synechococcus* sp. PCC 7002 and *Cyanothece* sp. PCC 7424 and in consent with those reports (Schirmer et al., 2010).

### Transcriptional organization of alkane synthesis genes in *Synechocystis* 6803

**Figure 2:**
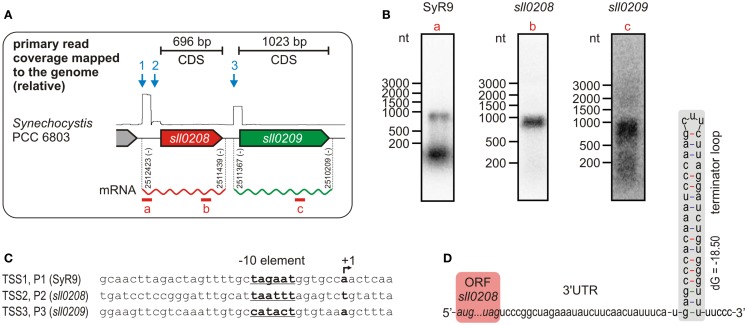
**Transcriptional organization of the gene cluster encoding acyl-ACP reductase (*aar*, *sll0209*) and aldehyde deformylating oxygenase (*ado*, *sll0208*) in *Synechocystis* sp. PCC 6803**. **(A)** Differential RNA sequencing (dRNAseq, data shown as primary read coverage mapped to the corresponding genomic region) of cells grown under standard conditions revealed independent transcriptional start sites (TSS) for both genes indicating for monocistronic mRNAs. The rectangular shape within the graph results from the enrichment of primary 5′-PPP carrying transcripts representing a TSS and the read length (101 nt) of the sequence analysis. Data were extracted from Kopf et al., 2014. Each TSS is represented by an arrow. The experimentally confirmed 5′ and 3′ ends of the main mRNAs are given by the respective genomic positions. **(B)** Northern verification for the monocistronic mRNAs and the small transcript SyR9, which accumulates independently from the *sll0208* mRNA in *Synechocystis* sp. PCC 6803. The part covered by the labeled probe is shown by a red bar in **(A)**. Consistent with the TSS mapping all transcripts detected appeared too short for a dicistronic mRNA. **(C)** Putative promoter elements upstream of the mapped TSS which is designated as +1. **(D)** The mapped 3′ end of the *sll0208* mRNA can be folded into a stem loop secondary structure that is typical for a Rho-independent terminator of transcription.

Data so far available suggest that *ado* and *aar* orthologs form an operon, but a genome-wide mapping of TSS in *Synechocystis* 6803 by using differential RNA sequencing (dRNAseq) revealed that both genes possess their own, specific, TSS (Mitschke et al., 2011a). Thus, they do not seem to be part of an operon since transcription is driven by independent promoters (Figure 2A). This unexpected transcriptional organization was further substantiated by northern blots (Figure 2B). Hybridization of an RNA probe specific for the *ado* (*sll0208*) ORF, which has a length of 696 bp, yielded a signal of about 900–1000 nt. This size is too short for a dicistronic mRNA, for which a minimal length of >1700 nt would be expected. Moreover, with an *aar*-specific probe, again, a transcript too short for a dicistron was detected but which approximately matches with the length of the *aar* (*sll0209*) open reading frame (1023 bp). Thus, these hybridization signals are consistent with the generation of independent monocistronic mRNAs from two independent promoters. Interestingly, our data uncovered not only two independent TSS for *ado* and *aar* but also a third TSS, which might belong to a putative non-coding RNA, called SyR9, upstream of the two genes (Mitschke et al., 2011a). This finding is supported by the identification of suitable −10 elements within the putative promoter sequences associated with these three TSS (Figure 2C). Hybridization using a SyR9-specific RNA probe yielded two signals – the lower band corresponds to the small transcript of SyR9, the upper band, however, was of approximately the same size as found for the *ado* transcript (Figure 2B). In this case, the upper signal could represent a combined RNA that included SyR9 and the *ado* mRNA. To test this possibility, PCR was carried out on cDNA samples prepared from total RNA circularized by RNA ligase and using primers in outbound orientation. One primer was located within the SyR9 segment and the other at the end of the coding region of *ado*. The obtained fragments were sequenced and revealed the 5′ end mapped for SyR9 to be connected to a sequence finishing 75 nt behind the last nucleotide of the *ado* stop codon [at genomic pos. 2511439 (complement)]. Thus, this analysis confirmed the SyR9-*ado* cotranscript and yielded its precise length of 985 nt, consistent with the sizes estimated by northern analysis. We conclude that in *Synechocystis* expression of *ado* is driven by two promoters, P1 and P2, and that the smaller accumulating transcript SyR9 results from processing of the SyR9–mRNA cotranscript or alternative termination of transcription.

The same approach was taken to determine the precise 5′ and 3′ ends of the *aar* mRNA. Whereas the 5′ end 72 nt upstream of the start codon resulting from initiation of transcription at promoter P3 was confirmed, its 3′ end was mapped 64 nt downstream of the stop codon [at genomic pos. 2510209 (complement)]. Thus, the total length of the *aar* mRNA is 1159 nt, consistent with the major signal obtained in the northern hybridization (Figure 2B) and the accumulation of this mRNA as a monocistronic transcript species. This finding receives further strong support from the identification of a putative Rho-independent terminator mapped here at the 3′ end of the *ado* mRNA (Figure 2D). We conclude that the *ado* and *aar* orthologs in *Synechocystis* 6803 are not transcriptionally organized as an operon. These results clearly impact approaches to manipulate the expression of these genes, as the activity of a strong promoter upstream of a *ado*–*aar* two-gene-cassette will lead to a high expression of the first, but not the second gene.

### Transcriptional organization of alkane synthesis genes in other cyanobacteria

**Figure 3:**
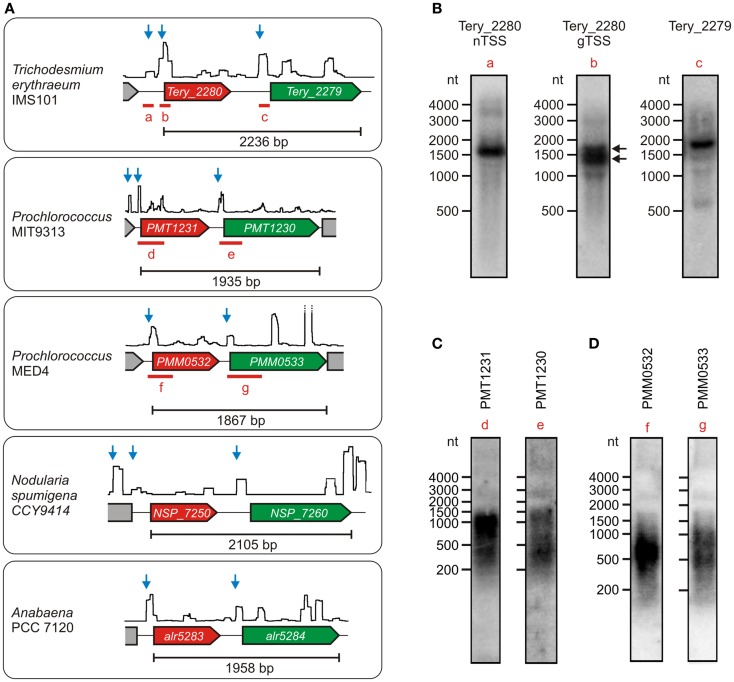
**Transcriptional organization of the gene cluster encoding *ado* (red) and *aar* (green) in various cyanobacteria under the respective standard conditions**. **(A)** The data are presented analogous to Figure 2. Each TSS potentially belonging to the respective *aar*/*ado* mRNA is represented by a blue arrow. Data were extracted from Mitschke et al. (2011b), Voß et al. (2013), Voigt et al. (2014), (Pfreundt et al., submitted). In each case, the minimum length of the dicistronic arrangement is given for the sequence between the start codon of *ado* and the stop codon of *aar*. **(B)** Northern verification of *ado* (Tery_2280) and *aar* (Tery_2279) mRNAs in *Trichodesmium erythraeum* IMS101. The black arrows indicate the two mRNAs originating from the independent TSS upstream of Tery_2280. **(C,D)** Respective northern blots for both mRNAs in *Prochlorococcus* sp. MIT9313 and MED4. The corresponding probe loci are given in **(A)**.

*Synechocystis* 6803 has been established as a representative model strain. Nevertheless, cyanobacteria constitute a physiologically and genomically very diverse taxon, hence the independent transcription of *ado* and *aar* found in *Synechocystis* might be different in other cyanobacteria. To test this possibility, we checked the primary transcriptomes that are available for several additional cyanobacteria (Mitschke et al., 2011b; Voß et al., 2013; Voigt et al., 2014; Pfreundt et al., submitted). Interestingly, independent TSS for the two genes exist also in *Anabaena* PCC 7120, *N. spumigena* CCY9414, *Prochlorococcus* MIT9313 & MED4, and *T. erythraeum* IMS101 (Figure 3A). The respective transcripts were exemplarily verified for three of these strains. In all cases, the main accumulating RNAs that derive from the TSS upstream of *ado* were shorter than the minimum length of a dicistronic transcript (Figures 3B–D). Moreover, the signal patterns that were observed after hybridization with *aar*-specific probes were different from those for the *ado* transcripts. These findings indicate that both genes might be transcriptionally independent from each other also in most other cyanobacteria, which is contrary to their seemingly dicistronic/polycistronic arrangement that appears largely conserved at the genomic level. Interestingly, similar to the arrangement in *Synechocystis* 6803, a second TSS upstream of *ado* is active in *Trichodesmium*, *Nodularia*, and *Prochlorococcus* MIT9313, which suggests that *ado* might be transcribed from two independent promoters also in several other species. For *Trichodesmium*, this arrangement was verified further by using a labeled probe specific for the sequence around the distal TSS (probe a, locus shown in Figure 3A). A transcript of ~1600 nt was detected, which demonstrates the existence of a long transcript that encompasses also the coding region of the *ado* gene (Figure 3B). Additionally, the two bands that were obtained when using a probe specific for a sequence downstream of the proximal *ado*-TSS (probe b) demonstrated the accumulation of two different mRNAs originating from the two TSS (indicated by black arrows).

### Experimental verification of the mapped *ado* and *aar* promoters in *Synechocystis* 6803

**Figure 4:**
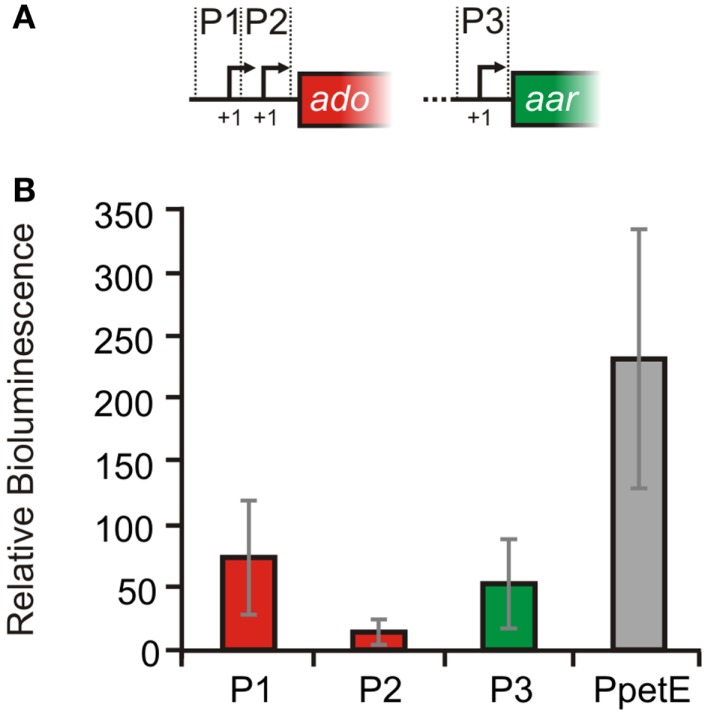
**Verification of promoter activities in *Synechocystis* sp. PCC 6803**. **(A)** Schematic illustration of the putative promoter regions. For the reporter assays *luxAB* genes were fused with the upstream sequences of either: SyR9 ( =P1, −116 to +2), *ado* ( =P2, −108 to +106 nt) or *aar* ( =P3, −401 to +71 nt), and also *petE* ( =P*petE*, −273 to +81, positive control) while +1 represents the first transcribed nucleotide. **(B)** Bioluminescence was measured *in vivo* as total light counts per second. A strain harboring a promoterless *luxAB* operon was used as negative control. The cultures were grown in standard BG11. For the P*petE* activity measurements, however, 2 μM CuSO_4_ was added. Relative bioluminescence was calculated by subtraction of the values obtained from negative control cultures. Data are the mean ± SD of 60 single measurements obtained with two biological replicates ( =independent transformants) in several independent experiments.

In order to experimentally verify the independent TSS for *ado* and *aar*, 5′ RACE experiments were performed with RNA extracted from *Synechocystis* 6803. All three start sites derived from the global mapping of TSS (Mitschke et al., 2011a), could be confirmed [TSS1 at pos. 2512423, TSS2 at pos. 2512315, TSS3 at pos. 2511367; positions are given for the complementary (fwd) strand]. Sequences upstream of the TSS contain putative −10 elements and were assumed as true promoters and therefore designated as P1 (distal *ado* promoter, SyR9 promoter), P2 (proximal *ado* promoter), and P3 (*aar* promoter, see Figure 2). In order to verify the suggested promoter activities, the sequences upstream and around the corresponding TSS were fused to *luxAB* luciferase genes. With respect to the first transcribed nucleotide (+1) we considered sequence ranges from −116 to +2 for P1, −108 to +106 nt for P2, and −401 to +71 for P3, respectively. Bioluminescence measurements revealed that these sequences indeed contain elements also driving transcription of the reporter gene (Figure 4). P1 and P2 showed about one third of the strength measured for the well-characterized promoter of the *petE* gene, which is highly active in the presence of copper ions and has been used for heterologous expression in *Anabaena* as well as *Synechocystis* (Buikema and Haselkorn, 2001; Tan et al., 2011). The data verified that the expression of *ado* and *aar* is regulated independently and that two promoters are found upstream of *ado*. Moreover, luminescence measured for P2 was close to background levels indicating that *luxAB* transcription initiated by this promoter is rather weak, consistent with the much lower read number observed for TSS2 in the dRNAseq analysis (Figure 2A).

### Expression of alkane synthesis genes in *Synechocystis* 6803

The described data suggest the autonomous regulation and possibly independent function of *ado* and *aar* genes under certain growth conditions. Thus, a detailed expression analysis could reveal if both are differentially expressed or even contrary regulated. Moreover, it might imply a functional involvement of the genes and possibly alkanes in adapting cyanobacterial physiology according to environmental changes since the function of alkanes in cyanobacteria is still ambiguous. To investigate if *ado* and *aar* are differentially expressed we compiled data extracted from CyanoEXpress, a database for microarray experiments performed with samples from *Synechocystis* (Hernandez-Prieto and Futschik, 2012). Interestingly, there are many conditions for which contrary fold changes for both genes were observed, further verifying their independent transcription. For the full list of fold changes see Table S5 in Supplementary Material. However, for the environmental conditions that are included in the CyanoEXpress database no clear and convincing stimulation of expression was evident. For *ado* the top log2 fold change (1.52) was observed after 4 h of zinc excess, whereas for *aar* it was 12 h of iron depletion (log2 fold change = 1.08). Nevertheless, diminished mRNA levels were observed for both, *ado* and *aar*, when cells were subjected to oxidative stress by H_2_O_2_ treatment. Furthermore, expression of both genes appear dependent on DNA gyrase since a novobiocin treatment in combination with heat stress, low temperature, or salt stress led to significantly reduced mRNA abundances (Table 1).

**Table 1:** **Log2 fold changes for *ado* and *aar* in *Synechocystis* 6803 after treatment with novobiocin in addition to heat stress, low temperature, and salt stress**.

Condition	*ado* (*sll0208*)	*aar* (*sll0209*)
WT_novobiocin	0.09	−0.16
WT_novobiocin_HS	−2.54	−2.24
WT_novobiocin_LT	−1.57	−0.10
WT_novobiocin_salt_stress	−1.16	−1.13

*Data were extracted from the CyanoEXpress database (Hernandez-Prieto and Futschik, 2012)*.

**Figure 5:**
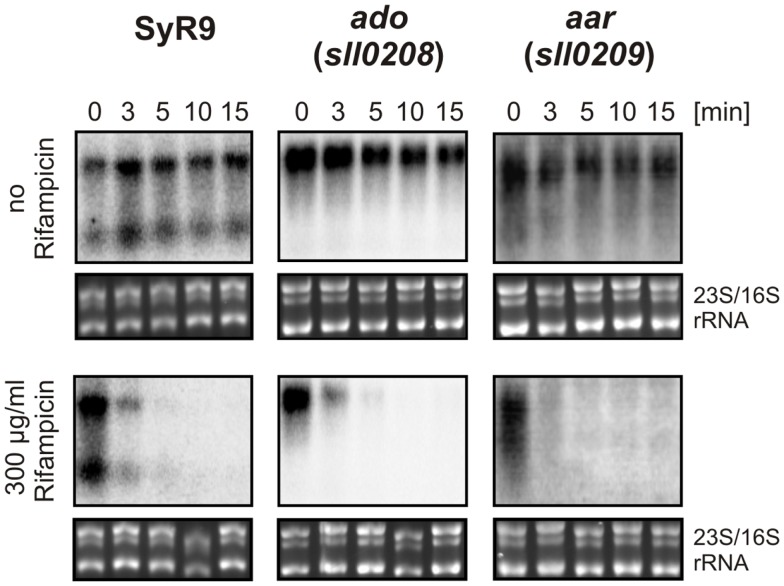
**RNA stability measurements**. Half-lives of the indicated transcripts were determined in the presence of rifampicin in cultures for the times indicated. For comparison, samples were taken at the identical time points but in the absence of rifampicin. For the control of equal loading, the large rRNA bands stained with ethidium bromide are shown underneath each hybridization.

The actual concentration of an mRNA is the result of two processes, transcription and turnover. Therefore, the stability of an mRNA is of similar importance for gene expression as is the control of transcription. To measure the relative transcript stability, we added the inhibitor of transcription initiation, rifampicin, to our cultures and then followed the disappearance of SyR9 and the mRNAs for *ado* and *aar* in a time course experiment (Figure 5). The calculated half-lives of all these transcripts were with <3 min quite short. We observed a transcript stability decreasing from SyR9 with a half-life of 2.2 min over 1.8 min for *ado* (*sll0208*), to only 1.6 min for *aar* (*sll0209*) at the end of this gene cluster in *Synechocystis* 6803.

## Discussion

**Figure 6:**
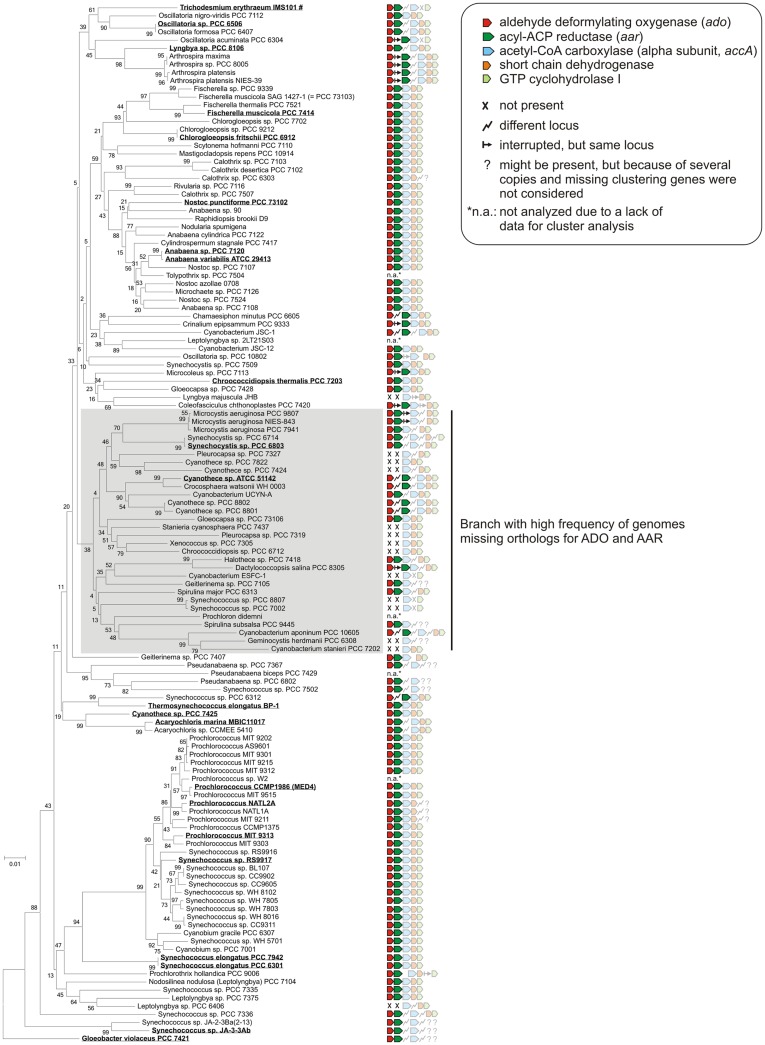
**Clustering of alkane biosynthesis genes among the cyanobacterial phylum**. The phylogenetic tree was generated by using the neighbor joining algorithm based on 16S rRNA sequences that were extracted from the SILVA database (Quast et al., 2013). Orthologs of aldehyde deformylating oxygenase and acyl-ACP reductase were searched by using blastP (threshold 1e^−5^) and the protein sequences from *Synechocystis* sp. PCC 6803 as reference. For the other three genes encoding acetyl-CoA carboxylase, short-chain dehydrogenase, GTP cyclohydrolase I the sequences from *Anabaena* sp. PCC 7120 were used. Strains for which alkane biosynthesis has been reported (Schirmer et al., 2010; Coates et al., 2014) are highlighted and underlined. Alkanes were measured either directly in cyanobacterial cell extracts (mainly penta- or hepta-decane) or indirectly by alkane detection in recombinant *E. coli* strains expressing the corresponding *ado* orthologs in combination with the *aar* gene from *S. elongatus* PCC7942. ^#^Alkanes were detected in a natural surface bloom of *Trichodesmium erythraeum* near Port Aransas in the Gulf of Mexico (Winters et al., 1969).

Since the discovery of the cyanobacterial alkane biosynthesis pathway, the major scientific focus has been on the enzymatic properties of ADO and AAR, as well as their potential for biofuel production (Schirmer et al., 2010; Li et al., 2011, 2012; Zhang et al., 2013). Less attention was paid to the expression of the corresponding genes as well as their regulation and possible function in the host organisms. Obviously, in cyanobacteria no other pathways (at least for medium-chain alkanes) exist since a knockout of *ado* and *aar* genes abolished heptadecane synthesis in *Synechocystis* 6803 and no alkanes were detected in *Synechococcus* sp. PCC 7002, a strain that lacks these two genes naturally (Schirmer et al., 2010). Due to the wealth of new genomic information that has become available within the last 2 years, comparative analyses linking phylogeny and the presence of alkane biosynthesis genes has become straightforward and is yielding new insight. Consistently, orthologs of ADO and AAR were found in all strains for which alkane detection has been reported earlier (see Figure 6). Additionally, alkanes were also detected in many other cyanobacterial isolates for which no genome sequence is available, including the genera *Oscillatoria*, *Microcoleus*, *Lyngbya*, *Nostoc*, *Plectonema*, *Chlorogloeopsis*, and *Phormidium* (Han et al., 1968; Winters et al., 1969), indicating that *ado* and *aar* genes are also present in those species. Surprisingly, orthologs of ADO and AAR are not present in other prokaryotes, associating this pathway to cyanobacteria or certain aspects of their phototrophic life style.

### Multiple promoters associated with the alkane biosynthesis genes in cyanobacteria

The clustering of *ado* and *aar* genes is widely conserved throughout the cyanobacterial phylum and frequently three additional genes are found associated with them (Figure 6). Despite their close functional connection and co-appearance in most genomes, our data demonstrate that in all tested strains the *ado* and *aar* genes are transcribed from separate, specific promoters. Thus, these two genes can be expressed independently from each other, in turn enabling their different or even divergent regulation. Due to the presence of separate regulatory elements, the dominance of monocistronic mRNAs and additionally the fact that the two genes are split in some species we speculate that physiological situations might exist for which an independent transcription of *ado* and *aar* could be advantageous. Additionally, in some strains *ado* is transcribed from two separate TSS, which increases the transcriptional complexity further. Multiple TSS may indicate different functions. In *Synechocystis*, for instance, two separate TSS were also reported for the *petH* gene that encodes two isoforms of ferredoxin:NADP oxidoreductase, while the decision between both isoforms is triggered by the differential use of these two TSS (Omairi-Nasser et al., 2011). Under standard conditions a proximal promoter is active, resulting in a short 5′UTR whereas under nitrogen depletion a more distally located promoter is active that mediates the transcription of *petH* with a long 5′UTR. Under both conditions only one isoform – a long or a short – is dominant. The different translation initiation sites are dictated by alternative secondary structures only depending on the length of the 5′UTR (Omairi-Nasser et al., 2011). In principle, alternative translation initiation is also imaginable for the *ado* gene. For the transcription of *ado* under standard growth conditions the distal promoter P1 appeared as the dominant regulatory element but situations might exist when the main expression is mediated by P2. Two mRNA species hence offer the possibility of alternative RNA foldings, which could then affect translation initiation or the yield of translated protein.

A highly interesting aspect of the observation that *ado* and *aar* genes are transcribed from separate and distinct promoters in cyanobacteria are the possible metabolic implications. Their monocistronic layout found in this work allows the regulatory autonomy of these two genes. Thus, they may signify also other, unknown, pathways branching off from aldehyde synthesis that would require their separate and non-stoichiometric expression. Such pathways might, e.g., lead to free fatty acid (from aldehyde oxidation) or even fatty alcohols (from aldehyde reduction) for which separate regulation of these two genes would be required. So, it is quite possible that still other unknown aldehyde-derived pathways remain to be discovered in cyanobacteria.

### Genomic organization of alkane synthesis genes in cyanobacteria

The initial step of alkane synthesis produces a fatty aldehyde by the conversion of a fatty acyl-ACP thus connecting the pathway to fatty acid metabolism. Interestingly, in many cyanobacteria *ado* and *aar* are found adjacent to *accA* encoding the alpha subunit of acetyl-CoA carboxylase (ACCase, EC 6.4.1.2). ACCase catalyzes the synthesis of malonyl-CoA, which is the first and rate-limiting step in fatty acid synthesis (Figure S1 in Supplementary Material). Accordingly, overexpression of ACCase in *Synechocystis* leads to an enhancement of hydrocarbon production especially heptadecane (Tan et al., 2011). Moreover, in *Anabaena* 7120 and also most other strains the gene downstream of *accA* encodes a protein that possibly belongs to the short-chain dehydrogenase/reductase (SDR) family of NAD- or NADP-dependent oxidoreductases (Joernvall et al., 1995). Interestingly, it shows high similarity to the 3-oxoacyl-[acyl-carrier-protein] reductase (EC 1.1.1.100) that also participates in fatty acid biosynthesis. However, despite the clustering and the functional relation, the *accA* gene possesses its own TSS in several strains, similar to the transcriptional separation of *ado* and *aar* (see Figure 3A, accumulating primary transcripts at the 3′ end of the *aar* gene), indicating a possibly independent regulation. The functional connection, if any, to the fifth and last gene in this cluster (Figure 6), encoding GTP cyclohydrolase I (EC 3.5.4.16), is less obvious. This enzyme is involved in folate biosynthesis from GTP and during the catalysis formate is generated. There is one parallel as during the conversion of the fatty aldehyde to the final alkane catalyzed by ADO, also formate is generated (Li et al., 2011), but if that has any meaning is currently unknown.

### Regulation of alkane synthesis gene expression

It is interesting to note the short half-lives of <3 min under our standard growth conditions, which we observed for all three major transcripts originating from the *syR9/ado/aar* locus. Consequently, slight changes in transcript stability could impact the mRNA accumulation and therefore the expression of these genes. Theoretically, a differential expression of *ado* and *aar* might point to conditions under which alkanes are physiologically more relevant. However, examining the wealth of expression data available for *Synechocystis* we found no clear evidence for differences in the abundance of *ado* or *aar* mRNA that could be linked to differences in the growth conditions. Expression may well be regulated in a multi-factorial fashion and not exclusively dependent on a single stimuli. Alternatively, the synthesis rate of alkanes could also be regulated by other factors than at the level of transcription or transcript stability, e.g., by modulating enzyme activities. Nevertheless, a regulatory cascade targeting *ado* and *aar* might exist but the stimuli remain ambiguous. Interestingly, most of the strains lacking AAR and ADO orthologs, and most probably alkanes, appear on one branch of the phylogenetic tree (Figure 6). Assuming a general importance of alkane synthesis, it is tempting to speculate that their physiological function in some strains of this clade might have been replaced or compensated by other mechanisms or compounds. Indeed, in these strains a likely compensatory polyketide synthase pathway which produces 1-alkenes is present (Coates et al., 2014).

### Physiological functions of cyanobacterial alkane synthesis

Although the presence of ADO and AAR appears to be restricted to cyanobacteria, alkanes were also detected in other prokaryotes (Ladygina et al., 2006), where different types of enzymes must be responsible for their biosynthesis. Indeed, alkanes are widely distributed throughout nature, including plants (Cheesbrough and Kolattukudy, 1984; Bernard et al., 2012), green algae (Dennis and Kolattukudy, 1991), and higher animals (Cheesbrough and Kolattukudy, 1988), where they are mainly a component of surface waxes and function as water barrier or in insects where they serve as pheromones (Blomquist and Jackson, 1979; Howard and Blomquist, 2005). Therefore, the physiological function of alkanes is obviously not restricted to cyanobacteria and a more general role can be assumed also for other prokaryotes. Since fatty acid synthesis is mainly necessary for membrane generation, alkanes might also have an influence on membrane composition and fluidity under particular conditions. However, the functional relevance of alkanes under optimal growth conditions might be low since the pathway can be deleted without any obvious growth phenotype (Schirmer et al., 2010) and due to the fact that 10% of all cyanobacterial genomes lack the ADO–AAR pathway altogether.

Cyanobacteria are equipped with several mechanisms to deal with superfluous electrons generated from excess light energy absorbed. Therefore, it might appear as an attractive additional mechanism if newly synthesized alkanes would serve as electron sinks under certain conditions. In such a scenario, one would expect massive accumulation of alkanes under suitable conditions, e.g., high light stress. To the best of our knowledge this has not been observed in wildtype strains thus far, rendering this possibility highly speculative. For instance, the heptadecane content in various cyanobacteria typically ranges between 0.02 and 0.1% of cell dry weight (Coates et al., 2014). For *Synechocystis* 6803, amounts of ~200 μg/L (0.13% of cell dry weight) were reported (Wang et al., 2013), which is relatively low compared to dominant molecules such as chlorophyll a (~8 mg/L), glycogen (~200 mg/L), or the osmoprotectant glucosylglycerol [~100 mg/L, representative values for cells of *Microcystis firma* shocked with 770 mM NaCl, (Erdmann et al., 1992)]. However, for genetically engineered *Synechocystis* strains it was also shown that redirecting the carbon flux to acyl-ACP and overexpressing alkane biosynthetic genes simultaneously can significantly increase the yield of heptadecane to 26 mg/L (1.1% of cell dry weight; Wang et al., 2013). Indeed, these data indicate that alkane accumulation is basically possible.

Nevertheless, the complex transcriptional organization of the alkane synthesis gene cluster needs to be taken into account when manipulating the expression of these genes *in situ*. For example, the insertion of strong or controllable promoters upstream of the *ado* (*sll0208*) gene in *Synechocystis* 6803 is very likely to have an effect on the transcription of this but not of the downstream located *aar* gene due to the presence of the Rho-independent terminator of transcription in between.

## Author Contributions

Wolfgang R. Hess designed the study. Stephan Klähn and Wolfgang R. Hess supervised the research. Stephan Klähn performed genomic analyses. Claudia Steglich and Desirée Baumgartner performed verification experiments for the promoter mapping. Desirée Baumgartner executed physiological experiments, Northern blots, and promoter analyses for *Synechocystis*. Ulrike Pfreundt and Karsten Voigt performed northern verification for *Trichodesmium* and *Prochlorococcus*, respectively. Verena Schön generated the decanal producing host strain for luciferase reporter assays in *Synechocystis*. Stephan Klähn and Wolfgang R. Hess evaluated and interpreted the data. Stephan Klähn, Desirée Baumgartner, and Wolfgang R. Hess wrote the manuscript.

## Conflict of Interest Statement

The authors declare that the research was conducted in the absence of any commercial or financial relationships that could be construed as a potential conflict of interest.

## Supplementary Material

The Supplementary Material for this article can be found online at http://www.frontiersin.org/Journal/10.3389/fbioe.2014.00024/abstract
