# Supplementary material for: Alkane Biosynthesis Genes in Cyanobacteria and Their Transcriptional Organization
Source: Front Bioeng Biotechnol. 2014 Jul 14;2:24. doi: 10.3389/fbioe.2014.00024 (PMC4094844; doi:10.3389/fbioe.2014.00024)
Supplement: Supplementary file 1 [file DataSheet_1.ZIP › Figure S1.pdf]

Supplementary figures

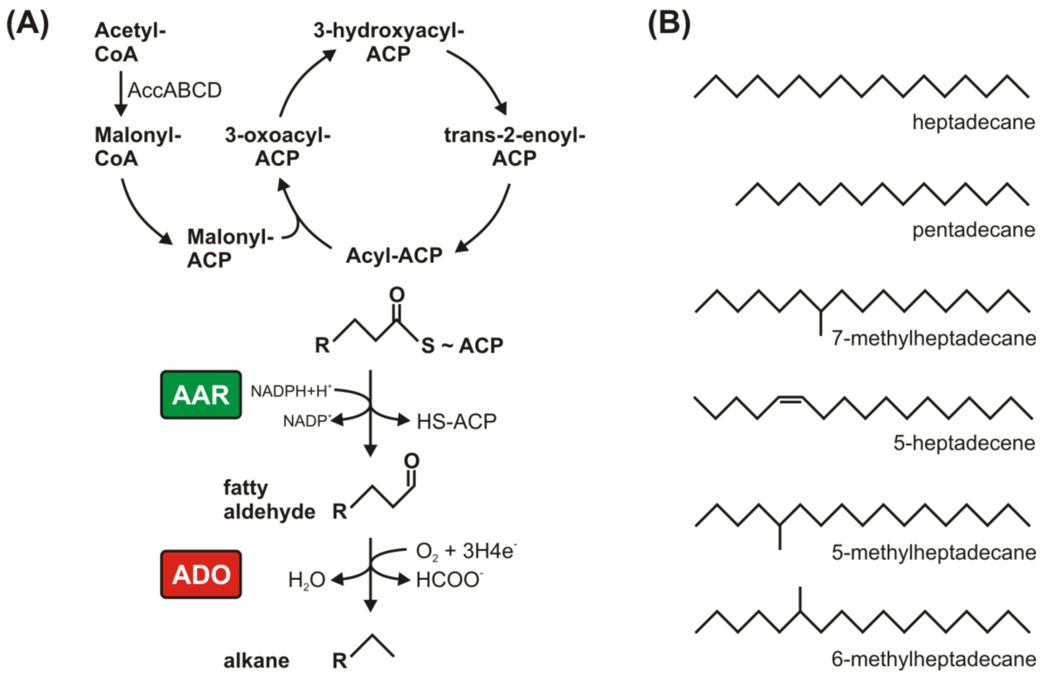

Fig. S1: (A) Schematic illustration of the ADO/AAR pathway which removes acyl-ACP precursors from the chain elongation cycle of fatty acid synthesis. **(B)** Alkanes that were reported for strains harboring the ADO/AAR pathway. In most strains heptadecane was the dominant hydrocarbon (Schirmer et al., 2010). *Lyngbya* sp. PCC 8106 is the only strain reported which possesses the AAR/ADO pathway but produced alkenes which probably derives from a monounsaturated fatty acid (Coates et al., 2014). However, recently it was shown that strains lacking *ado* and *aar* are also able to produce hydrocarbons via a polyketide synthase pathway. These strains (including *Synechococcus* sp. PCC 7002) mainly synthesize 1-alkenes (not shown here, Coates et al., 2014).
